# Supplementary material for: Does it work? Using a Meta-Impact score to examine global effects in quasi-experimental intervention studies
Source: PLoS One. 2022 Mar 17;17(3):e0265312. doi: 10.1371/journal.pone.0265312 (PMC8929616; doi:10.1371/journal.pone.0265312)
Supplement: S1 Appendix — (DOCX) [file pone.0265312.s001.docx]

**S1 Appendix:** *Coaching content protocol for one-to-one and group conditions*

| **Week and Topics covered^a^** | **Coaching Methods** |
| --- | --- |
| **Week 1:** **Why are we here?**  Overview of research and purpose  Meeting each other  Setting outcomes  Thinking about thinking  Remembering that we remember well!  Notice your internal world – which senses you use to think in?  Developmental task:  Think of a time or an example of something you remember well | Visual and oral presentation (group) discussion.  Development of metacognitive awareness through silence, observation and discussion [1] |
| **Week 2:** **Natural Strategies for Memory**  How do we remember when we remember well?  What state do we need to be in to remember well?  Memory for names  Memory for space  Memory for image  Memory for sound  Memory for numbers  Developmental task:  Practice a strategy at least 3 times. | Metacognitive exercises such as ‘Kim’s Game’ [2] de-briefed (in groups and pairs) with clean questions [3]. |
| **Week 3: How do we Think about Time?**  Using Space and Time to remember  Developmental Task: note your current time management tools such as diaries and electronic reminders, how to these fit with your models for time? | ‘Clean Space’, metacognitive exercise for resolving conflicts between desired outcomes and practical realities [4]. |
| **Week 4: Emotional Resilience**  Strategies for concentration and paying attention (taking things in)  Developmental Task: practice moving from ‘drama’ to ‘calmer’ | ‘Triune Brain’ and ‘Drama Triangle’, two models for understanding how our emotional states are affected by, and can be improved via, physiology [5] and relationships at work [6]. |
| **Week 5:** **Strategies**  Time and organisation  Putting into context    Developmental task: put into practice your outcomes from today | Goal Setting [7] and Clean Space [4]. |
| **Week 6**: Any Remaining Issues!  Reflecting on what we have learned and what we will do next | (Pair and group) de-briefing using Clean Questions [8], [9] and Clean Feedback [10]. |

^A^ Information from this column was presented as notes to participants

References

[1] S. Coutinho and G. Neuman, “learning style and self-efficacy,” *Learn. Environ. Res.*, vol. 11, pp. 131–151, 2008, doi: 10.1007/s10984-008-9042-7.

[2] R. Fisher, “Still thinking: The case for meditation with children,” *Think. Ski. Creat.*, vol. 1, no. 2, pp. 146–151, 2006, doi: 10.1016/j.tsc.2006.06.004.

[3] P. Tosey, J. Lawley, and R. Meese, “Eliciting Metaphor through Clean Language: An Innovation in Qualitative Research,” *Br. J. Manag.*, vol. 25, no. 3, pp. 629–646, 2014.

[4] J. Lawley and A. I. Manea, “The Use of Clean Space to Facilitate a ‘ Stuck ’ Client – a Case Study,” *J. Exp. Psychother.*, vol. 20, no. 4, pp. 62–70, 2017, [Online]. Available: http://jep.ro/images/pdf/cuprins_reviste/80_art_7_v2.pdf.

[5] S. Palmer, “Leaders : Own Your Alligator Brain,” *Public Manag.*, pp. 68–70, 2013.

[6] R. C. Burgess, “A Model for Enhancing Individual and Organisational Learning of ‘Emotional Intelligence’: The Drama and Winner’s Triangles,” *Soc. Work Educ.*, vol. 24, no. 1, pp. 97–112, 2005, doi: 10.1080/0261547052000325008.

[7] J. Whitmore, *Coaching for Performance*. London: Nicholas Brealey Publishing Limited, 1992.

[8] C. Walker, *From Contempt to Curiosity*. Fareham, UK: Clean Publishing, 2014.

[9] N. Doyle, P. Tosey, and C. Walker, “Systemic Modelling: Installing Coaching as a Catalyst for Organisational Learning,” *E-Organisations & People,* vol. 17, no. 4, 2010.

[10] B. Walsh, S. Nixon, C. Walker, and N. Doyle, “Using a Clean Feedback Model to Facilitate the Learning Process,” *Creat. Educ.*, vol. 6, pp. 953–960, 2015, doi: 10.4236/ce.2015.610097.
