# Supplementary material for: Does it work? Using a Meta-Impact score to examine global effects in quasi-experimental intervention studies
Source: PLoS One. 2022 Mar 17;17(3):e0265312. doi: 10.1371/journal.pone.0265312 (PMC8929616; doi:10.1371/journal.pone.0265312)
Supplement: S2 Appendix — (DOCX) [file pone.0265312.s002.docx]

**S2 Appendix:** *Z-scores and MI score per participant for CS1 (abbreviations as in S2 Table)*

| Condition (1 = one-to-one, 2 = control, 3 = group coaching | WMRS | PS Job perf | WM Job perf | Stress | Digit span | GSES | Number of Improvements per participant |
| --- | --- | --- | --- | --- | --- | --- | --- |
| 1 |  |  |  |  | -0.94 |  | 0 |
| 1 |  |  |  |  | 1.1 |  | 1 |
| 1 |  |  |  |  | -0.43 |  | 0 |
| 1 | 0.37 | 0.3 | 0.63 | 0.34 | 2.13 | -1 | 1 |
| 1 | 1.19 | 0.02 | -0.55 | 0.34 | 0.08 | -0.13 | 1 |
| 1 | 1.19 | 0.02 | -0.55 | 0.34 | 0.08 | -0.13 | 1 |
| 1 | 3.44 | 0.88 | 2.01 | 0.34 | 0.08 | -1.38 | 2 |
| 1 | 0.47 | 1.45 | 1.42 | 1.29 | 1.36 | -0.63 | 4 |
| 1 | 0.27 | 1.45 | -0.16 | -0.6 | -0.43 | -0.25 | 1 |
| 1 | 0.27 | 0.59 | 1.61 | 1.29 | -0.94 | -0.63 | 3 |
| 1 | -0.35 | -1.42 | -1.34 | -0.6 |  | 0.13 | 0 |
| 1 | -0.86 | -0.27 |  | -0.6 | 0.08 | -0.75 | 0 |
| 1 | -1.17 | 0.3 | 0.04 | 0.34 | -1.19 | -0.38 | 0 |
| 1 | 0.78 | -0.56 | -0.94 | -0.6 | -0.94 | -0.25 | 0 |
| 1 | 0.23 | -0.56 | -0.16 | -0.6 | -0.43 | -0.63 | 0 |
| 1 |  |  |  |  | -0.43 |  | 0 |
| 2 | -0.65 | 0.02 | -0.94 | -0.6 | -0.43 | 0.25 | 0 |
| 2 | -0.86 | -1.13 | -1.92 | -0.6 | -1.96 | 0.13 | 0 |
| 2 |  |  |  |  | 0.08 |  | 0 |
| 2 |  | 0.3 | -0.16 | -1.55 | 0.08 | -0.38 | 0 |
| 2 |  |  |  |  | -2.47 |  | 0 |
| 2 |  |  |  |  | 0.59 |  | 0 |
| 2 | -0.25 | -0.27 | -0.75 | 0.34 | 0.34 | -0.63 | 0 |
| 2 | -0.04 | -2.56 |  | -0.6 | 0.59 | 0 | 0 |
| 2 | -0.96 | -0.84 | -0.55 | 0.34 | -0.94 | -0.13 | 0 |
| 2 | -1.68 | -1.7 | -1.34 | -0.6 | -0.94 | 0.5 | 0 |
| 2 |  |  |  |  | 0.59 |  | 0 |
| 2 | -1.47 | -0.27 | -0.94 | -0.6 | 1.36 | 0.5 | 1 |
| 3 | 0.78 | 0.88 | 1.02 | 1.29 | -1.45 | -0.63 | 2 |
| 3 |  | 0.3 | 0.24 | -2.5 |  | -0.75 | 1 |
| 3 |  | 2.02 | 1.81 | 2.24 | 0.59 | 1.5 | 4 |
| 3 | 0.27 | -0.27 | 0.43 | 1.29 | 0.59 | 0.13 | 1 |
| 3 | -0.25 | 0.88 | -0.16 | -0.6 | 0.59 | 0 | 0 |
| 3 | -0.45 | 0.3 | 0.63 | 0.34 | -0.17 | -0.88 | 0 |
| 3 | 1.29 | 1.16 |  | 0.34 |  | -0.5 | 2 |
| 3 | 0.78 | -0.27 |  | 1.29 | 0.59 | 0.25 | 1 |
| 3 |  |  |  |  | 1.1 |  | 1 |
| 3 | 0.37 | 0.3 | 1.22 | -0.6 | 1.1 | -0.63 | 2 |
| 3 |  |  |  |  | 1.36 |  | 1 |
| 3 | -1.06 | -0.84 | 0.04 | 0.34 | -0.43 | 0 | 0 |
| 3 | 0.47 | 1.16 | 0.04 | 1.29 | -0.17 | -0.63 | 2 |
| 3 | 0.06 | -1.42 | 0.04 | 0.34 | -0.43 | -0.38 | 0 |
| 3 | -0.65 | -0.56 | -1.14 | -1.55 | 0.08 | 0 | 0 |
