# Supplementary material for: Does it work? Using a Meta-Impact score to examine global effects in quasi-experimental intervention studies
Source: PLoS One. 2022 Mar 17;17(3):e0265312. doi: 10.1371/journal.pone.0265312 (PMC8929616; doi:10.1371/journal.pone.0265312)
Supplement: S3 Appendix — (DOCX) [file pone.0265312.s003.docx]

**S3 Appendix:** *Z-scores and MI score per participant for CS2 (abbreviations as in S3 Table)*

| Condition (1=intervention, 2= control) | Digit span | Memstrat | Memcap | Memanx | Mem  Achieve | Mem ctrol/se | WSE | Number of Improvements per participant |
| --- | --- | --- | --- | --- | --- | --- | --- | --- |
| 1 | 0.89 | 0.18 | 1.93 | 1.06 | 0.65 | 0.54 | 0.68 | 2 |
| 1 | -0.87 | 0.35 | 0.15 | 1.04 | 1.18 | 1.01 | 0.68 | 3 |
| 1 | -0.87 | -1.9 | 1.21 | 1.72 | -1.75 | 1.01 | 0.98 | 3 |
| 1 | 1.47 | -0.46 | 1.91 | 1.06 | -1.49 | 2.21 | 1.87 | 5 |
| 1 | 2.06 | 0.18 | -1.26 | -0.58 | 0.11 | 1.49 | 1.87 | 3 |
| 1 | -0.87 | 0.97 | -0.22 | -0.6 | -0.17 | 0.54 | 0.68 | 0 |
| 1 | -0.28 | 0.49 | 0.85 | -0.26 | -0.42 | -0.18 | -2.01 | 0 |
| 1 | -0.28 | 0.97 | -2.34 | -2.24 | -0.42 | -3.29 | -2.01 | 0 |
| 1 | 0.89 | -1.56 | 0.15 | 0.39 | 1.18 | 0.54 | -0.22 | 1 |
| 1 | -1.45 | 0.32 | -0.92 | 0.39 | 0.11 | -0.66 | 0.38 | 0 |
| 1 | -1.45 | -0.13 | 0.87 | -0.91 | 0.37 | 0.06 | 0.08 | 0 |
| 1 | -0.87 | 0.01 | 1.21 | 0.39 | 0.11 | 0.77 | 1.27 | 2 |
| 1 | 0.89 | 0.18 | -0.58 | -1.92 | 1.46 | 0.77 | 0.38 | 1 |
| 1 | 1.47 | 0.18 | 0.15 | -0.26 | 1.18 | 0.54 | -1.12 | 2 |
| 1 | 2.06 | -0.77 | 0.15 | -0.91 | -0.7 | -0.9 | -1.12 | 1 |
| 1 | 0.89 | 0.66 | 0.15 | 0.39 | 1.18 | -0.18 | -0.82 | 1 |
| 1 | -0.28 | 1.78 | 0.51 | -1.25 | -0.7 | 1.01 | -0.22 | 2 |
| 1 | -1.45 |  |  |  |  |  | -1.12 | 0 |
| 1 | 0.3 | 0.35 | -0.19 | -0.26 | 0.11 | -1.38 | -1.71 | 0 |
| 1 | -0.87 | 0.18 | 0.51 | -0.6 | 0.9 | 0.3 | 1.27 | 1 |
| 1 | 0.3 | -0.44 | 0.49 | 1.4 | 0.11 | 1.73 | 1.27 | 3 |
| 1 | 1.47 | 2.9 | -0.92 | -0.6 | 2 | -1.14 | -1.41 | 3 |
| 1 | 1.47 | -1.56 | 0.51 | 1.04 | 1.72 | 0.54 | 0.68 | 3 |
| 1 | 0.3 | 0.01 | -0.92 | 0.08 | 0.9 | -0.9 | -0.52 | 0 |
| 1 | 0.3 | 0.01 | -0.22 | -1.25 | 0.65 | -0.9 | 1.57 | 1 |
| 1 | -0.28 | 0.66 | 0.51 | 1.04 | -0.17 | 0.77 | 0.38 | 1 |
| 2 | 0.3 | 2.09 | 0.85 | 1.06 | 0.11 | 1.25 | 1.27 | 4 |
| 2 | -0.87 | -0.46 | 0.51 | 1.06 | -1.77 | -1.14 | -1.12 | 1 |
| 2 | -0.28 | -0.13 | -0.22 | 0.08 | 0.39 | -0.18 | -0.22 | 0 |
| 2 | -0.87 | -2.21 | -0.22 | 2.7 | -1.49 | 0.3 | 0.38 | 1 |
| 2 | -0.28 |  |  |  |  |  | 0.08 | 0 |
| 2 | 0.3 | -0.13 | 0.15 | -0.26 | 1.18 | -0.9 | -0.22 | 1 |
| 2 | 0.89 |  |  |  |  |  |  | 0 |
| 2 | -0.28 | -0.3 | 1.21 | 0.39 | -0.14 | -0.42 | 0.08 | 1 |
| 2 | -1.45 | -0.63 | 0.49 | -1.25 | 0.37 | -0.42 | 0.08 | 0 |
| 2 | -0.28 |  |  |  |  |  |  | 0 |
| 2 | 0.89 |  | -1.98 | -0.4 | -0.42 | 0.06 | 0.38 | 0 |
| 2 | 0.89 |  |  |  |  |  | 0.08 | 0 |
| 2 | -0.87 | -1.73 | 0.51 | 0.73 | -1.77 | -1.62 | -0.82 | 0 |
| 2 | 0.3 | -0.13 | -1.28 | 0.39 | -0.42 | -0.9 | -1.41 | 0 |
| 2 | -2.04 | -1.9 | -3.04 | -0.6 | -2.31 | -0.18 |  | 0 |
| 2 | 0.89 | 0.8 | -0.92 | -0.91 | 1.44 | 0.3 | -0.82 | 1 |
| 2 | 0.3 | 0.35 | 0.51 | 0.06 | -0.42 | 1.25 | 0.68 | 1 |
| 2 | -0.28 |  |  |  |  |  | 1.27 | 1 |
| 2 | 0.3 | 0.49 | -0.56 | -1.92 | -0.17 | -0.42 | -0.82 | 0 |
| 2 | 0.3 | 0.01 | -0.22 | 0.39 | -0.42 | 0.06 | 0.08 | 0 |
| 2 | -1.45 | 0.35 | 0.51 | -0.93 | -0.96 | 0.06 | 0.38 | 0 |
| 2 | -1.45 | 0.66 | -0.92 | -0.26 | -0.42 | -0.66 | 0.38 | 0 |
| 2 | -0.87 | -0.77 | -0.56 | 0.08 | -0.96 | 0.3 | 0.68 | 0 |
| 2 | -0.28 | -0.3 | 0.15 | 0.39 | -0.42 | -0.9 | -1.12 | 0 |
| 2 | 1.47 | -0.3 | 0.51 | 0.73 | -0.14 | -0.66 | -1.12 | 1 |
| 2 | -0.28 | 0.66 | 0.87 | 0.08 | 0.65 | 0.54 | 0.08 | 0 |
