# Supplementary material for: Does it work? Using a Meta-Impact score to examine global effects in quasi-experimental intervention studies
Source: PLoS One. 2022 Mar 17;17(3):e0265312. doi: 10.1371/journal.pone.0265312 (PMC8929616; doi:10.1371/journal.pone.0265312)
Supplement: S4 Appendix — (DOCX) [file pone.0265312.s004.docx]

**S4 Appendix:** *Actual score cut off points delineated by the >/= +1 SD dichotomization*

| Variable | Range | Number above the mean |
| --- | --- | --- |
| CS1 WMRS (behaviour) | 0-3 | 0.76 |
| CS1 WM related Job Performance | 1-5 | 1.29 |
| CS1 SE related Job Performance | 1-5 | 1.24 |
| CS1 Stress | 1-5 | 1.69 |
| CS1 SE | 1-5 | 0.28 |
| CS2 Memory Strategy (Behavioural) | 1-5 | 0.54 |
| CS2 Memory Capacity (Behavioural) | 1-5 | 0.4 |
| CS2 Memory Anxiety (Emotional) | 1-5 | 0.64 |
| CS2 Memory Achievement | 1-5 | 0.41 |
| CS2 Memory SE | 1-5 | 0.59 |
| CS2 Workplace SE | 1-5 | 0.51 |
