# Supplementary material for: Does it work? Using a Meta-Impact score to examine global effects in quasi-experimental intervention studies
Source: PLoS One. 2022 Mar 17;17(3):e0265312. doi: 10.1371/journal.pone.0265312 (PMC8929616; doi:10.1371/journal.pone.0265312)
Supplement: S5 Appendix — (DOCX) [file pone.0265312.s005.docx]

**S5 Appendix:**

*Sample Items and Internal Consistency Analyses for the Dependent Variables*

| Test | Publisher | Inter-item correlation | Alpha-coefficient | Sample item / explanation |
| --- | --- | --- | --- | --- |
| CS1 | | | | |
| Working memory behaviour rating scale (WMRS) (Alloway, Gathercole, & Kirkwood, 2008) | (90) | .35 | .901 | 0-3 scale with 0 representing ‘not typical at all’ and 3 representing ‘very typical’. For example: “requires regular repetition of instructions” |
| Job performance self-ratings related to cognitive skills (WM Job Perf) | (91) | .31 | .77-.87 | How confident do you feel (1-5) regarding, for example: “planning and prioritizing your time; keeping track of what you have to do.” |
| Job performance self-ratings related to psycho-social skills (PS Job Perf) | (91) | .42 | .77-.87 | How confident do you feel (1-5) regarding, for example: “asking for emotional support; knowing your strengths.” |
| Single item rating of stress level | (95) | n/a | .77-.87 | How confident do you feel (1-5) at managing stress |
| General Self Efficacy (GSES) | (96) | .81-87  (test-retest = .81) | > .8 | 1-5 Likert scale. For example: “I often feel like a failure” and “I can handle the situations that life brings.” |
| Backwards digit span | (51) | Test-retest 0.69-0.71  Alpha co-efficient .82 | | Numbers are recited verbally and the participant must recall the numbers in reverse order with no errors or omissions. |
| CS2 | | | | |
| Backward digit span | (51) | Test-retest 0.69-0.71  Alpha co-efficient .82 | | Numbers are recited verbally and the participant must recall the numbers in reverse order with no errors or omissions. |
| Memory Strategies (MemStrat) | (92) | .37 | .84 | 1-5 Likert scale. For example: “Do you post reminders of things you need to do in a prominent place to remind you?” |
| Memory Capacity (MemCap) | (92) | .29 | .79 | 1-5 Likert scale. For example: “I am good at remembering names.” |
| Memory Anxiety (MemAnx) | (92) | .58 | .90 | 1-5 Likert scale. For example: “I do not get flustered when I am put on the spot to remember new things.” |
| Memory control SE  (MemCtrlSE) | (92) | .4 | .85 | 1-5 Likert scale. For example: “If I were to work on my memory, I could improve it.” |
| Memory Achievement  (MemAchieve) | (92) | .4 | .87 | 1-5 Likert scale. For example: “I work hard at trying to improve my memory.” |
| Workplace SE  (WSE) | (94) | .58-.78 | > .82 | Thinking about your work, how well can you (1-5 Likert Scale) for example: “achieve goals that will be assigned.” |
