# Supplementary figures and images for: Does it work? Using a Meta-Impact score to examine global effects in quasi-experimental intervention studies
Source: PLoS One. 2022 Mar 17;17(3):e0265312. doi: 10.1371/journal.pone.0265312 (PMC8929616; doi:10.1371/journal.pone.0265312)

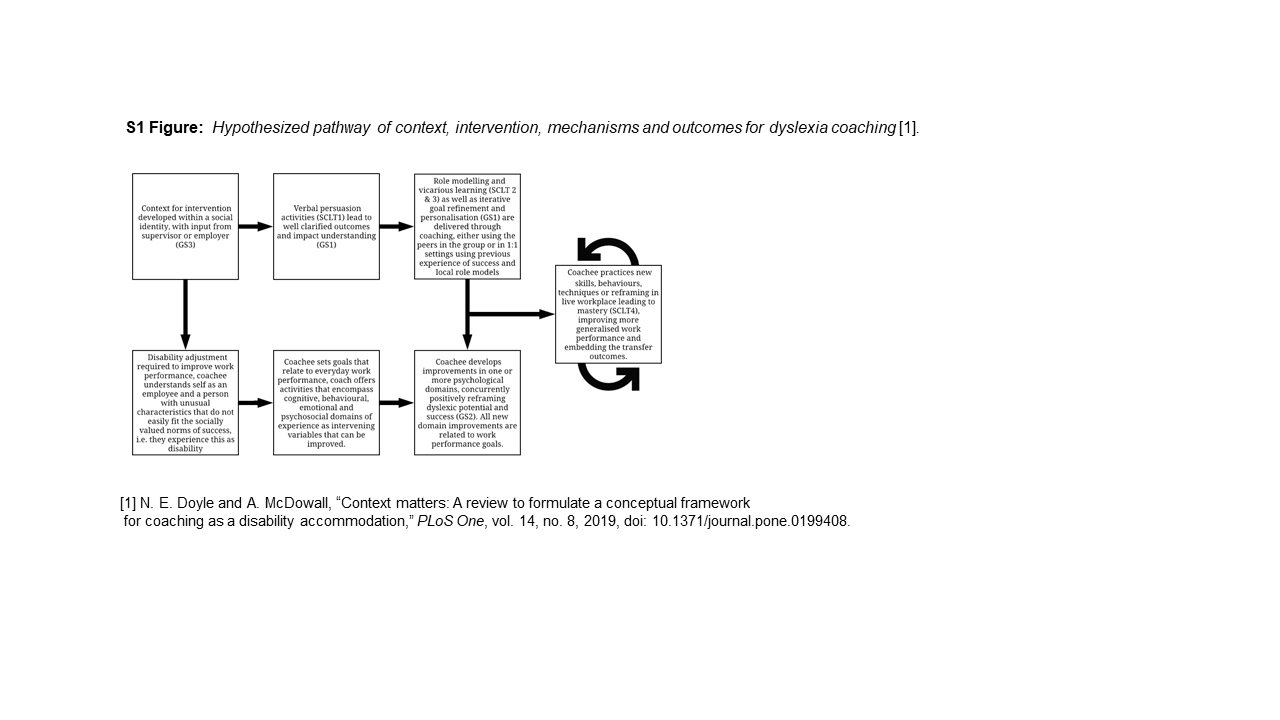

Supplement: S1 Fig — (TIF) [file pone.0265312.s006.tif]

**S1 Graph:** *Working Memory Standard Scores per group per time interval, CS1*


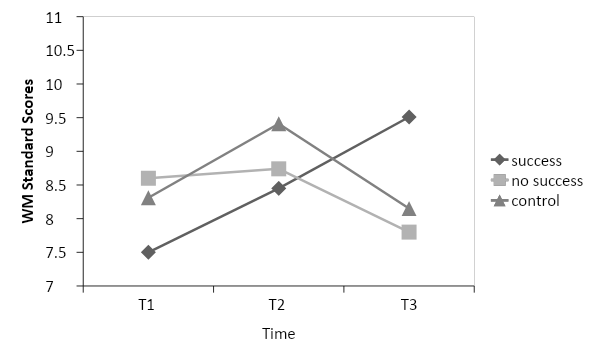

Supplement: S1 Graph — (DOCX) [file pone.0265312.s007.docx]
