# Supplementary material for: Does it work? Using a Meta-Impact score to examine global effects in quasi-experimental intervention studies
Source: PLoS One. 2022 Mar 17;17(3):e0265312. doi: 10.1371/journal.pone.0265312 (PMC8929616; doi:10.1371/journal.pone.0265312)

**S2 Graph:** *Meta-Impact means and confidence intervals for CS1*

*(1=one-to-one; 2=control; 3=group coaching. Circles/asterisk = outliers)*


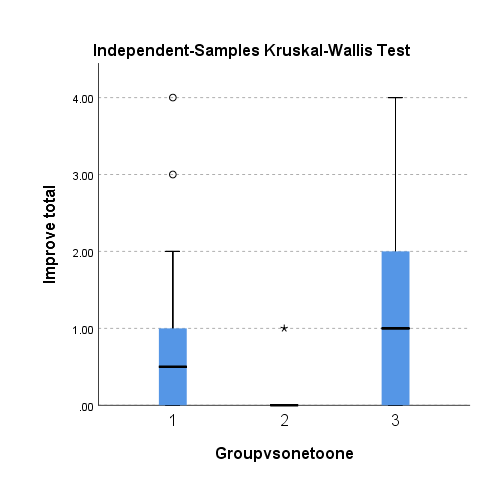

Supplement: S2 Graph — (DOCX) [file pone.0265312.s008.docx]
