# Supplementary material for: Does it work? Using a Meta-Impact score to examine global effects in quasi-experimental intervention studies
Source: PLoS One. 2022 Mar 17;17(3):e0265312. doi: 10.1371/journal.pone.0265312 (PMC8929616; doi:10.1371/journal.pone.0265312)
Supplement: S1 Table — (DOCX) [file pone.0265312.s009.docx]

**S1 Table:** *Study* *domains and outcome measures*

| Domain | CS1 | CS2 |
| --- | --- | --- |
| Cognitive | Backward digit span [1] | Backward digit span [1] |
| Behavioural | Working Memory (WM) Behaviour Rating scale (WMRS) [2]  Job Performance self-ratings related to WM skills [3]  Job Performance self-ratings related to psycho-social communication [3] | Memory Strategies [4]  Memory Capacity [4] |
| Emotional | Single item rating of stress level | Memory-related Anxiety [4] |
| Psycho-social | General Self-Efficacy (SE) [5] | Memory Control SE [4]  Memory Achievement SE [4]  Workplace SE [6] |

**References**

[1] D. Weschler, *Weschler Adult Intelligence Scale version IV*. San Antonio, Texas: Pearson, 2008.

[2] T. P. Alloway, S. E. Gathercole, and H. Kirkwood, *The Working Memory Rating Scale*. London: Pearson Assessment, 2008.

[3] N. Doyle, “A Critical Realist Analysis of Coaching as a Disability Accommodation,” City, University of London, 2018.

[4] R. A. Dixon and D. F. Hultsch, “The Metamemory in Adulthood (MIA) instrument.,” *Psychol. Doc.*, vol. 14, no. 3, 1984.

[5] Judge, T. A., E. A. Locke, C. C. Durham, and A. N. Kluger, “Dispositional effects on job and life satisfaction: The role of core evaluations.,” *J. Appl. Psychol.*, vol. 83, pp. 17–34, 1998.

[6] S. J. Pepe, M. L. Farnese, F. Avalone, and M. Vecchione, “Work Self-Efficacy Scale and Search for Work Self-Efficacy Scale : A Validation Study in Spanish and Italian Cultural Contexts,” *Rev. Psicol. del Trab. y las Organ.*, vol. 26, no. 3, pp. 201–210, 2010, doi: 10.5093/tr2010v26n3a4.
