# Supplementary material for: Does it work? Using a Meta-Impact score to examine global effects in quasi-experimental intervention studies
Source: PLoS One. 2022 Mar 17;17(3):e0265312. doi: 10.1371/journal.pone.0265312 (PMC8929616; doi:10.1371/journal.pone.0265312)
Supplement: S2 Table — (DOCX) [file pone.0265312.s010.docx]

**S2 Table:** *Means and standard deviations for CS1*

| Measure* | 1:1 | | | Group | | | Control | | |
| --- | --- | --- | --- | --- | --- | --- | --- | --- | --- |
|  | **T1**  ***N=***  ***M* (*SD*)** | **T2**  ***N=***  ***M* (*SD*)** | **T3**  ***N=***  ***M* (*SD*)** | **T1**  ***N=***  ***M* (*SD*)** | **T2**  ***N=***  ***M* (*SD*)** | **T3**  ***N=***  ***M* (*SD*)** | **T1**  ***N=***  ***M* (*SD*)** | **T2**  ***N=***  ***M* (*SD*)** | **T3**  ***N=***  ***M* (*SD*)** |
| Digit Span  (Cognitive) | *n=*22  7.95 (1.53) | *n=*15  8.03 (2.29) | *n=*14  8.93  (1.60) | *n=*23  7.96 (2.03) | *n=*19  8.74 (3.40) | *n=*19  9.34 (2.10) | *n=*22  8.31 (1.76) | *n=*16  9.41 (2.43) | *n=*13  8.15 (1.92) |
| WMRS  (Behavioural) | *n=*21  1.38 (0.67) | *n=*16  1.06 (0.46) | *n=*11  0.81 (0.31) | *n=*22  1.39 (0.48) | *n=*16  1.00 (0.45) | *n=*13  0.96 (0.45) | *n=*19  1.28 (0.47) | *n=*16  1.22 (0.40) | *n=*9  1.35 (0.40) |
| GSES  (Psycho-social) | *n=*21  2.26 (0.43) | *n=*16  2.11 (0.63) | *n=*11  1.73 (0.45) | *n=*22  2.62 (0.63) | *n=*16  2.24 (0.62) | *n=*15  2.39 (0.71) | *n=*19  2.46 (0.52) | *n=*15  2.38 (0.72) | *n=*8  2.52 (0.57) |
| Job Perf. (WM related behaviour) | *n=*21  2.88 (.65) | *n=*16  3.53  (.51) | *n=*10  3.71 (.49) | *n=*20  2.79 (.62) | *n=*16  3.55 (.55) | *n=*15  3.51 (.42) | *n=*18  2.99 (.43) | *n=*16  3.09  (.52) | *n=*9  3.07  (.35) |
| Job Perf (Psychosocial related behaviour) | *n=*21  2.98 (.78) | *n=*16  3.49  (.88) | *n=*11  4.00 (.38) | *n=*22  2.99 (.69) | *n=*16  3.88 (.77) | *n=*15  3.65 (.81) | *n=*19  2.86  (.70) | *n=*16  3.19 (.80) | *n=*9  2.91  (.54) |
| Stress Mgmt (Emotional) | *n=*18  2.86 (1.01) | *n=*11  3.25 (0.93) | *n=*8  3.55 (0.82) | *n=*22  2.63 (1.36) | *n=*16  3.5 (1.32) | *n=*15  3.1 (0.99) | *n=*19  3.32 (1.00) | *n=*16  3.13 (1.02) | *n=*9  3.00 (1.22) |

*****(see appendix 2 for scoring ranges)
