# Supplementary material for: Does it work? Using a Meta-Impact score to examine global effects in quasi-experimental intervention studies
Source: PLoS One. 2022 Mar 17;17(3):e0265312. doi: 10.1371/journal.pone.0265312 (PMC8929616; doi:10.1371/journal.pone.0265312)
Supplement: S3 Table — (DOCX) [file pone.0265312.s011.docx]

**S3 Table:**  *Means and standard deviations for CS2*

| Measure* | Group | | | Control | | |
| --- | --- | --- | --- | --- | --- | --- |
|  | **T1**  ***N=***  ***M* (*SD*)** | **T2**  ***N=***  ***M* (*SD*)** | **T3**  ***N=***  ***M* (*SD*)** | **T1**  ***N=***  ***M* (*SD*)** | **T2**  ***N=***  ***M* (*SD*)** | **T3**  ***N=***  ***M* (*SD*)** |
| Digit Span  (Cognitive) | *n=*26  7.731  (1.756) | *n=*26  8.769  (1.986) | *n=*26  9.539  (2.611) | *n=*26  8.077  (2.018) | *n=*26  8.654  (2.449) | *n=*26  9.231  (2.405) |
| Memory Strategies  (Behavioural) | *n=*26  3.743  (0.650) | *n=*26  3.783  (0.527) | *n=*25  3.909  (0.483) | *n=*23  3.865  (0.422) | *n=*20  3.810  (0.561) | *n=*23  3.815  (0.447) |
| Memory Capacity  (Behavioural) | *n=*26  3.102  (0.782) | *n=*26  3.202  (0.650) | *n=*25  3.133  (0.599) | *n=*24  3.090  (0.496) | *n=*20  3.200  (0.523) | *n=*23  3.015  (0.51) |
| Memory Anxiety  (Emotional) | *n=*26  2.039  (0.684) | *n=*26  2.132  (0.654) | *n=*25  2.133  (.555) | *n=*24  1.934  (0.581) | *n=*20  1.925  (0.621) | *n=*23  2.133  (0.648) |
| Memory Achievement  (Psychosocial) | *n=*26  3.974  (0.471) | *n=*26  4.066  (0.467) | *n=*25  4.072  (0.518) | *n=*24  3.973  (0.472) | *n=*20  3.729  (0.481) | *n=*23  3.673  (0.380) |
| Memory Self-efficacy  (Psychosocial) | *n=*26  3.356  (0.558) | *n=*26  3.979  (0.541) | *n=*25  3.858  (0.457) | *n=*24  3.438  (0.567) | *n=*20  3.500  (0.556) | *n=*23  3.475  (0.292) |
| Workplace Self-efficacy  (Psychosocial) | *n=*26  3.696  (0.612) | *n=*26  3.846  (0.638) | *n=*25  3.920  (0.728) | *n=*24  3.821  (0.479 | *n=*20  3.740  (0.674) | *n=*23  3.745  (0.614) |

(see appendix 3 for scoring ranges)
