# Supplementary material for: Does it work? Using a Meta-Impact score to examine global effects in quasi-experimental intervention studies
Source: PLoS One. 2022 Mar 17;17(3):e0265312. doi: 10.1371/journal.pone.0265312 (PMC8929616; doi:10.1371/journal.pone.0265312)
Supplement: S4 Table — (DOCX) [file pone.0265312.s012.docx]

**S4 Table:** *Group and time-wise comparisons for example variable from each study domain*

| Variable | Sample Size | Analysis | *β* power | *p* result | Effect size |
| --- | --- | --- | --- | --- | --- |
| CS1 cognitive | T1=67, T2=50, T3=46 | RM ANOVA  Time  Groups | *β=*.301  *β=*.242 | *p*=.231  *p*=.541 | *η²=*.038*  *η²=*.041* |
| CS1 Behavioural (WMRS) | T1=59, T2=48, T3=34 | RM ANOVA  Time  Groups | Not available | *p*<.001**  *p*=.042* | *η²=*.378***  *η²=*.217*** |
| CS1 Emotional | T1=59, T2=43, T3=32 | RM ANOVA  Time  Groups | Not available | *p*<.001**  *p*=.541 | *η²=*.279***  *η²=*.17*** |
| CS1 Self-efficacy | T1=67, T2=47, T3=34 | 1-way ANOVA T3 | *β=*.21 | *p*=.009** | *d=*1.26*** |
| CS2 cognitive | T1=52, T2=52, T3=52 | RM ANOVA  Time  Groups | *β=*1.0  *β=*.252 | *p*<.001**  *p*=.314 | *η²=*.313***  *η²=*.023* |
| CS2 Behavioral (Mem Strat) | T1=49, T2=44, T3=48 | RM ANOVA  Time  Groups | *β=*.341  *β=*.181 | *p*<.001**  *p*=.314 | *η²=*.041*  *η²=*.022* |
| CS2 Emotional | T1=50, T2=44, T3=48 | RM ANOVA  Time  Groups | *β=*.287  *β=*.077 | *p=*.238  *p*=.808 | *η²=*.039*  *η²=*.005 |
| CS2 Self-efficacy  (Mem Self-Efficacy) | T1=50, T2=44, T3=48 | RM ANOVA  Time  Groups | *β=*.962  *β=*.755 | *p*<.001**  *p*=.014* | *η²=*.193***  *η²=*.112** |
| *p values with * denotes significant at 0.05 level and with ** denotes significance at the <.01 level*  *η² values with *denote small effect size, ** denote medium and ***denote large* | | | | | |
