# Supplementary material for: Does it work? Using a Meta-Impact score to examine global effects in quasi-experimental intervention studies
Source: PLoS One. 2022 Mar 17;17(3):e0265312. doi: 10.1371/journal.pone.0265312 (PMC8929616; doi:10.1371/journal.pone.0265312)
Supplement: S5 Table — (DOCX) [file pone.0265312.s013.docx]

**S5 Table:** *Correlational analysis for CS1 raw changes*

| Domain | | GSES | Behavioural | Emotional | Cognitive |
| --- | --- | --- | --- | --- | --- |
| GSES | Pearson Corr. | 1 |  |  |  |
|  | Sig. (2-tailed) |  |  |  |  |
|  | *N* | 33 |  |  |  |
| Behavioural | Pearson Corr. | -0.348 | 1 |  |  |
|  | Sig. (2-tailed) | 0.065 |  |  |  |
|  | *N* | 29 | 29 |  |  |
| Emotional | Pearson Corr. | 0.119 | **.556**** | 1 |  |
|  | Sig. (2-tailed) | 0.509 | **0.002** |  |  |
|  | *N* | 33 | **29** | 33 |  |
| Cognitive | Pearson Corr. | -0.032 | 0.321 | 0.042 | 1 |
|  | Sig. (2-tailed) | 0.867 | 0.102 | 0.824 |  |
|  | *N* | 30 | 27 | 30 | 46 |
