# Supplementary material for: Does it work? Using a Meta-Impact score to examine global effects in quasi-experimental intervention studies
Source: PLoS One. 2022 Mar 17;17(3):e0265312. doi: 10.1371/journal.pone.0265312 (PMC8929616; doi:10.1371/journal.pone.0265312)
Supplement: S6 Table — (DOCX) [file pone.0265312.s014.docx]

**S6 Table:** *Correlation analysis for CS2 raw changes*

| **Domain** | | **Behavioural** | **Emotional** | **Memory SE** | **Cognitive** |
| --- | --- | --- | --- | --- | --- |
| **Behavioural** | Pearson Corr. | 1 |  |  |  |
|  | *N* | 45 |  |  |  |
|  |  |  |  |  |  |
| **Emotional** | Pearson Corr. | **-.403^**^** | 1 |  |  |
|  | Sig. (2-tailed) | **0.006** |  |  |  |
|  | *N* | **45** | 46 |  |  |
|  |  |  |  |  |  |
| **Memory SE** | Pearson Corre. | -0.030 | **.350^*^** | 1 |  |
|  | Sig. (2-tailed) | 0.844 | **0.017** |  |  |
|  | *N* | 45 | **46** | 46 |  |
|  |  |  |  |  |  |
| **Cognitive** | Pearson Corr. | 0.171 | -0.024 | 0.154 | 1 |
|  | Sig. (2-tailed) | 0.262 | 0.872 | 0.308 |  |
|  | *N* | 45 | 46 | 46 | 52 |
