# Supplementary material for: Does it work? Using a Meta-Impact score to examine global effects in quasi-experimental intervention studies
Source: PLoS One. 2022 Mar 17;17(3):e0265312. doi: 10.1371/journal.pone.0265312 (PMC8929616; doi:10.1371/journal.pone.0265312)
Supplement: S7 Table — (DOCX) [file pone.0265312.s015.docx]

**S7 Table:** *Descriptive z-score statistics and Meta-Impact scores, CS1*

|  | **One-to-one coaching** | | | | **Group coaching** | | | **Control** | | | | **Number of people with significant improvements per variable**  **(n from control group)** |
| --- | --- | --- | --- | --- | --- | --- | --- | --- | --- | --- | --- | --- |
|  | ***N*** | ***Mean*** | ***SD*** | ***N*** | | ***Mean*** | ***SD*** | | ***N*** | ***Mean*** | ***SD*** |  |
| **Cognitive *z-score*** | 15 | -0.05 | 0.94 | 13 | | 0.26 | 0.78 | | 12 | -0.26 | 1.13 | 7 (1) |
| **Behav scale 1 (WMRS) *z-score*** | 12 | 0.49 | 1.18 | 11 | | 0.15 | 0.70 | | 7 | -0.85 | 0.60 | 4 (0) |
| **Behav scale 2 (WM Job Performance related) *z-score*** | 12 | 0.18 | 0.84 | 13 | | 0.28 | 0.95 | | 8 | -0.81 | 0.96 | 6 (0) |
| **Behav scale 3 (Job Performance Communication related) *z-score*** | 11 | 0.18 | 1.09 | 11 | | 0.38 | 0.79 | | 7 | -0.94 | 0.57 | 5 (0) |
| **Emotional *z-score*** | 12 | 0.11 | 0.71 | 13 | | 0.27 | 1.31 | | 8 | -0.48 | 0.61 | 7 (0) |
| **Self-efficacy *z-score*** | 12 | -0.50 | 0.42 | 13 | | -0.19 | 0.63 | | 8 | 0.03 | 0.40 | 1 (0) |
| **MI Improve (condition mean)** | **16** | **0.88** | **1.20** | **15** | | **1.13** | **1.12** | | **12** | **0.08** | **0.29** | 8 out of 16 people from 121 and 10 out of 15 from group achieved at least one significant improvement.  Only one from the control achieved the same. |
| Note: A negative *z-*score for the condition x variable does not necessarily indicate no improvement; it may reflect a small or marginal improvement that is lower than the average improvement for the variable as a whole (see Appendix 3 for individual scores). | | | | | | | | | | | | |
