# Supplementary material for: Does it work? Using a Meta-Impact score to examine global effects in quasi-experimental intervention studies
Source: PLoS One. 2022 Mar 17;17(3):e0265312. doi: 10.1371/journal.pone.0265312 (PMC8929616; doi:10.1371/journal.pone.0265312)
Supplement: S8 Table — (DOCX) [file pone.0265312.s016.docx]

**S8 Table:** *Descriptive z-score statistics and Meta-Impact scores, CS2*

| Domain | Intervention | | | | Control | | | Number of people with significant improvements per variable  (n from control group) |
| --- | --- | --- | --- | --- | --- | --- | --- | --- |
|  | ***N*** | ***Mean*** | ***SD*** | ***N*** | | ***Mean*** | ***SD*** |  |
| Cognitive | 26 | 0.19 | 1.10 | 26 | | -0.19 | 0.87 | 7 (1) |
| Behav scale 1 (strategies) mean *z-*score | 25 | 0.14 | 1.00 | 20 | | -0.18 | 0.99 | 2 (0) |
| Behav scale 2 (capacity) mean *z-*score | 25 | 0.15 | 0.97 | 21 | | -0.17 | 1.03 | 5 (1) |
| Emotional mean *z-*score | 25 | -0.07 | 1.04 | 21 | | 0.08 | 0.97 | 9 (2) |
| SE Achievement mean *z-*score | 25 | 0.32 | 0.94 | 21 | | -0.39 | 0.95 | 8 (2) |
| SE Memory mean *z-*score | 25 | 0.17 | 1.17 | 21 | | -0.20 | 0.73 | 8 (2) |
| SE Workplace mean *z-*score | 26 | 0.07 | 1.19 | 23 | | -0.08 | 0.75 | 7 (1) |
| MI Improve mean | **26** | **1.46** | **1.36** | **26** | | **0.46** | **0.86** | 18 out of 26 from the intervention achieved at least one significant improvement, compared with 9 out of 26 from the control group. |
| Note: A negative *z-*score for the condition x variable does not necessarily indicate no improvement; it may reflect a small or marginal improvement that is lower than the average improvement for the variable as a whole (see Appendix 3 for individual scores). | | | | | | | | |
