# Supplementary material for: Does it work? Using a Meta-Impact score to examine global effects in quasi-experimental intervention studies
Source: PLoS One. 2022 Mar 17;17(3):e0265312. doi: 10.1371/journal.pone.0265312 (PMC8929616; doi:10.1371/journal.pone.0265312)
Supplement: S9 Table — (DOCX) [file pone.0265312.s017.docx]

**S9 Table:** *Person-specific examples from each case study*

|  | CS1 “Jane” | CS2 “John” |
| --- | --- | --- |
| Age | 47 | 58 |
| Education level | High School | Post-graduate |
| IQ | Verbal IQ in the superior range (standard score 16 within a 1-19 range) but processing speed and digit span scores below average (5 and 7 respectively, also on a 1-19 range) at T1. | IQ in the average range though higher for perceptual reasoning than for working memory by a significant amount [1]. |
| Experience | Jane experienced a large increase in her ability within the digit span from five to eleven at T2 and twelve at T3, which placed her in the top improvers with a *z-*score of 2.06. Jane did not have any other significant improvements, her other *z-*scores range from -1.12 to 0.15 which translate into real score differences (on scales of 1-5 where an improvement is positive) of between 0 and -.038. | John had a digit span score varying from 9 at T1, to 8 at T2 and then 10 at T3. However, he improved significantly in WMRS ratings and job performance; both within the behavioral domain (Table 1). His improvement *z*-scores for these were 3.44 and 2.01 respectively, which are very high, indicating that behavioral changes were the most impactful for John. |

**Reference**

[1] D. Weschler, *Weschler Adult Intelligence Scale version IV*. San Antonio, Texas: Pearson, 2008.
